# Lotus Leaf-Inspired Low-Adhesion Surface for a Droplet-Based Electricity Generator

**DOI:** 10.3390/biomimetics11090655

**Published:** 2026-09-12

**Authors:** Huachen Su, Yuying Yan

**Affiliations:** Faculty of Engineering, University of Nottingham, Nottingham NG7 2RX, UK; egxhs3@nottingham.ac.uk

**Keywords:** droplet-based electricity generator, FEP, surface wetting, partial immersion, lotus-inspired low-adhesion target, superhydrophobicity, energy harvesting

## Abstract

Droplet-based electricity generators (DEGs) are usually characterised under dry conditions, although retained water can alter their electrical boundary. We compared blank, bottom-wetted, top-wetted, wetted-and-dried, and partially immersed states using 40 mm × 40 mm FEP/ITO devices. Four separately assembled devices per condition were tested on a separate apparatus, with 100 droplet events per device. The device averages were analysed by Welch’s ANOVA and Holm-adjusted contrasts. Bottom wetting changed the average peak voltage by −0.30% relative to the blank (adjusted *p* = 0.960), whereas top wetting reduced it by 56.21% (adjusted *p* = 2.62 × 10^−9^). The dried-state average was within 0.38% of the blank (adjusted *p* = 0.960), indicating electrical recovery under the stated protocol without establishing equivalence or complete drying. Immersing 10%, 30%, and 50% of the device length reduced the average by 19.31%, 34.59%, and 47.73%, respectively. The common-state averages agreed within 1.49% with an earlier apparatus series. A process-matched MWCNT:FEP surface reached an apparent contact angle of 151° and a 6° sliding angle while retaining 87.3% of the FEP-only average. These results identify the exposed top surface, rather than the bottom-interface water, as the principal wetting-state sensitivity under the tested conditions and support a lotus-inspired low-adhesion design target.

## 1. Introduction

### 1.1. Droplet-Based Electricity Generation

Droplet-based electricity generators (DEGs) convert the kinetic and interfacial energy of impacting or sliding liquid into electrical pulses and have been proposed for rain-energy harvesting, self-powered sensing, and distributed monitoring on water-exposed structures [1,2,3,4,5,6,7,8,9]. In the common transistor-inspired architecture, liquid–solid contact electrification establishes interfacial charge, while droplet spreading and bridging of electrodes alter electrostatic induction and generate a transient external current [1,7,8,9,10,11,12,13,14]. The measured response, therefore, depends on both the charge-transfer interface and the time-dependent electrical boundary formed by the dielectric, electrodes, droplet, load, and measuring circuit.

Recent research has improved DEG output through dielectric selection, electrode geometry, surface patterning, droplet control, high-frequency operation, and power-management strategies [2,3,4,5,6,7,8,9]. Parallel studies of liquid–solid contact electrification show that electron transfer, ion adsorption, electric-double-layer formation, interfacial relaxation, and electrolyte concentration can all influence the observed signal; their relative importance remains system-dependent [10,11,12,13,15,16,17]. Superhydrophobic and low-adhesion designs can accelerate droplet departure and reduce liquid retention [17,18,19,20,21], but an increased apparent contact angle does not by itself isolate the electrical effect of residual water or establish a single charge-transfer mechanism.

### 1.2. Knowledge Gap and Study Rationale

A central limitation in the prevailing literature is that ‘wetting’, ‘residue’, and ‘immersion’ are often treated as a single disturbance even though they create physically different boundary conditions. A top-surface film directly replaces the initially dry liquid–FEP contact region; water trapped beneath the dielectric is separated from the active surface by a 0.20 mm FEP layer; and partial immersion introduces a remote conductive liquid boundary without necessarily wetting the impact zone. Comparisons that do not separate these locations cannot determine whether output loss originates mainly at the active contact interface or through a change in capacitive coupling, field distribution, or leakage [9,15,16,17,18].

A second limitation is the limited attention paid to recovery after a defined drying procedure. If the loaded output returns toward the blank-control level, irreversible gross loss of function becomes less consistent with the observation, but complete dehydration and unchanged surface properties still require independent verification. A location-resolved comparison combined with a wetted-and-dried specimen can, therefore, narrow, but not identify, plausible mechanisms more effectively than a dry-versus-wet comparison alone.

The practical relevance arises when the delivery of water exceeds drainage and evaporation, so that successive droplets encounter a pre-wetted rather than a dry surface. Such a state may occur during intense rain, splash exposure [Appendix A], condensation, operation at an orientation that inhibits drainage, or partial inundation. Humidity can extend liquid residence time, while orientation controls the gravitational driving force for shedding. However, the threshold conditions cannot be generalised from the present experiment because only 50% relative humidity and a 30° inclination were tested.

This study, therefore, separates blank-control, bottom-wetted, top-wetted, wetted-and-dried, and partial-immersion states within a matched DEG framework. The geometry-based working hierarchy is location dependent: (i) top-surface water is expected to produce the largest suppression because it directly changes the pre-impact state of the active interface; (ii) bottom-interface water may have a smaller effect, or one below the present detection limit, because the FEP separates it from the impacting droplet; (iii) partial immersion is expected to modify the remote coupled electrical boundary; and (iv) the loaded output may return toward the blank-control level after removal of visually apparent water if the electrical change is not permanent. Effective contact area, screening, leakage, capacitance, relaxation, dielectric hydration, and impact geometry remain competing explanations. This hierarchy does not assume that electric-double-layer formation is the sole mechanism.

### 1.3. Lotus-Inspired Low-Adhesion Target and Evidential Boundary

The biological model motivating the design target is the lotus leaf, whose hierarchical micro-/nanostructure and low-surface-energy wax chemistry are associated with a composite wetting state, low contact-line pinning, rapid droplet departure, and self-cleaning [22,23,24,25,26,27,28]. These characteristics have inspired water-repellent energy harvesters and structurally replicated DEGs [19,20,21]. In the present study, however, the abstraction is restricted to a low-adhesion water-shedding target; no lotus-like hierarchy or self-cleaning function is claimed because morphology and quantitative residue removal were not measured.

The engineering target is to maintain a sliding angle below the intended DEG operating inclination, reduce liquid residence between impacts, and preserve electrical functionality. Smooth FEP, with a measured 94° static contact angle, provides a mild-hydrophobicity baseline but does not establish low hysteresis or residue-free departure. The sprayed 1:10 MWCNT:FEP surface meets a preliminary lotus-inspired low-adhesion target through its 151° apparent static contact angle and 6° sliding angle for a 2 µL droplet [19,20,21]. These measurements do not demonstrate the lotus leaf’s hierarchical texture, residue-free shedding, or self-cleaning.

The testable biomimetic proposition is that a low-adhesion surface should reduce liquid residence and thereby lessen the wetting-state penalty during repeated operation. The present process-matched coating comparison tests DEG compatibility and wetting metrics, but it does not yet quantify multi-drop residue reduction or separate wettability from the electrical influence of MWCNT loading. Those points define the next validation step rather than an established conclusion.

### 1.4. Objective

Accordingly, this study quantifies how the location and extent of pre-existing water affect DEG peak voltage, evaluates electrical recovery under a defined drying protocol, examines representative pulse duration and external-load-branch energy, and evaluates a preliminary lotus-inspired, low-adhesion target-coating series. The specific contribution is a replicated location-resolved wetting map and a bounded mechanistic hierarchy. Its biomimetic contribution is functional rather than morphological: the lotus leaf’s low-adhesion water-shedding principle is translated into a measurable DEG surface target while compatibility with electrical output is assessed. The main wetting and immersion comparisons use independent-device replication; the auxiliary coating and surface-characterisation results remain descriptive.

## 2. Materials and Methods

### 2.1. DEG Architecture and Fabrication

**Figure 1:**
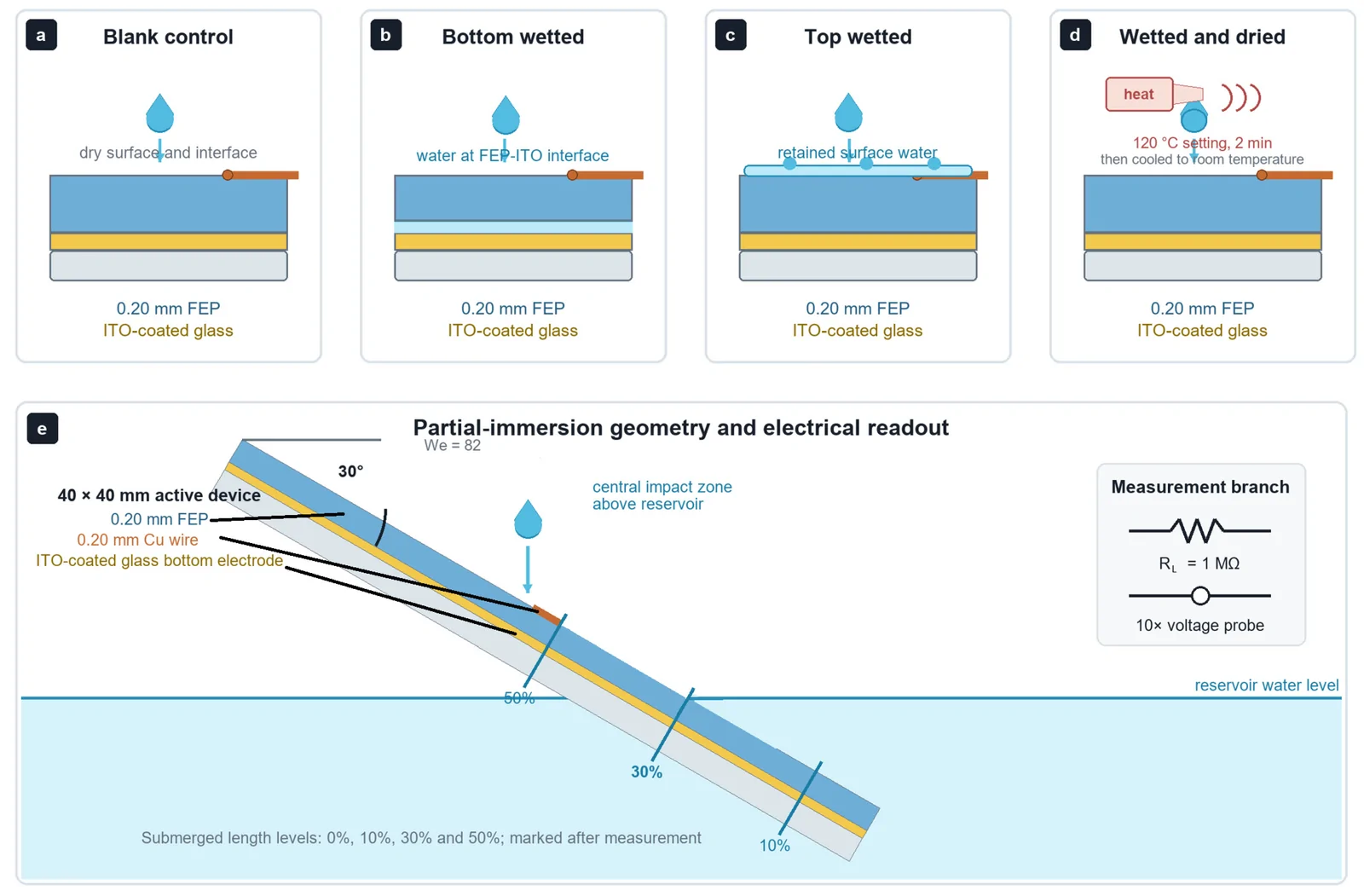
Device architecture and wetting configurations: (**a**) blank control; (**b**) bottom wetted; (**c**) top wetted; (**d**) wetted-and-dried; and (**e**) partial immersion with electrical readout.

Each DEG had a total footprint, FEP area, electroactive area, and ITO-coated area of 40 mm × 40 mm. The device consisted of a 0.20 mm fluorinated ethylene propylene (FEP) dielectric film on an indium tin oxide (ITO)-coated glass bottom electrode and a 0.20 mm-diameter copper-wire top electrode in the configuration shown schematically in Figure 1. The wire was positioned fully flush with the polymer surface and was retained by its own rigidity. The dielectric was DuPont Teflon FEP Film Type A, a copolymer film of tetrafluoroethylene and hexafluoropropylene that conforms to ASTM D3368 Type I [29].

Before assembly, the FEP and electrode components were cleaned by 15 min solvent soaking using acetone and ethanol, respectively. The components were stacked and bonded using a heat gun. The FEP received no charging, frictional treatment, electrical conditioning, or other pre-treatment. Four separately assembled devices were used for every main wetting and immersion condition, and no device was reused across conditions. The primary validation series was acquired on a separate apparatus from the preliminary experiments, using the operating settings described below, with 100 sequential droplet events recorded per device. The earlier same-laboratory series was retained only as a descriptive cross-apparatus check and was not pooled with the primary series.

### 2.2. Material and Surface Characterisation

The manufacturer datasheet reports nominal Type A FEP properties, including a dielectric constant of 2.15 at 25 °C and 1 kHz, a maximum dissipation factor of 0.0003, a minimum surface resistivity of 1 × 10^15^ Ω per square at 23 °C and 38% relative humidity, and a density of 2.13–2.17 g cm^−3^ at 23 °C [29]. These are manufacturer values for the product family, not measurements on the tested specimen. The tested film thickness was 0.20 mm.

A 2 µL deionised-water droplet was placed on each surface at 22 °C and measured with a contact-angle instrument. The archived dataset contains one analysable sessile-drop image per formulation; the reported values are, therefore, single representative apparent static measurements rather than replicate averages. Each image uses a horizontal baseline at the solid surface and tangent lines at the left and right three-phase contact points; the angle is measured through the liquid, consistent with standard sessile-drop practice [30]. The uncoated FEP film used in the wetting-location study gave 94°. No contact-angle hysteresis or sliding-angle result was available for that film, so this value establishes only mild static hydrophobicity.

### 2.3. Process-Matched MWCNT–FEP Low-Adhesion Target Coatings

All three comparison surfaces were prepared by the same FEP-powder spraying process, with only the MWCNT loading varied. The MWCNT:FEP powder ratios were 0:1 for the FEP-only control, 1:20 for the intermediate formulation, and 1:10 for the superhydrophobic formulation. The powders were dispersed in acetone, and the dispersion was preheated to 80 °C. An epoxy primer was sprayed onto the substrate; when the primer was semi-cured, the corresponding powder dispersion was sprayed over it. The coated samples were heated at 120 °C for 1 h and then conditioned at room temperature for 24 h.

**Figure 5:**
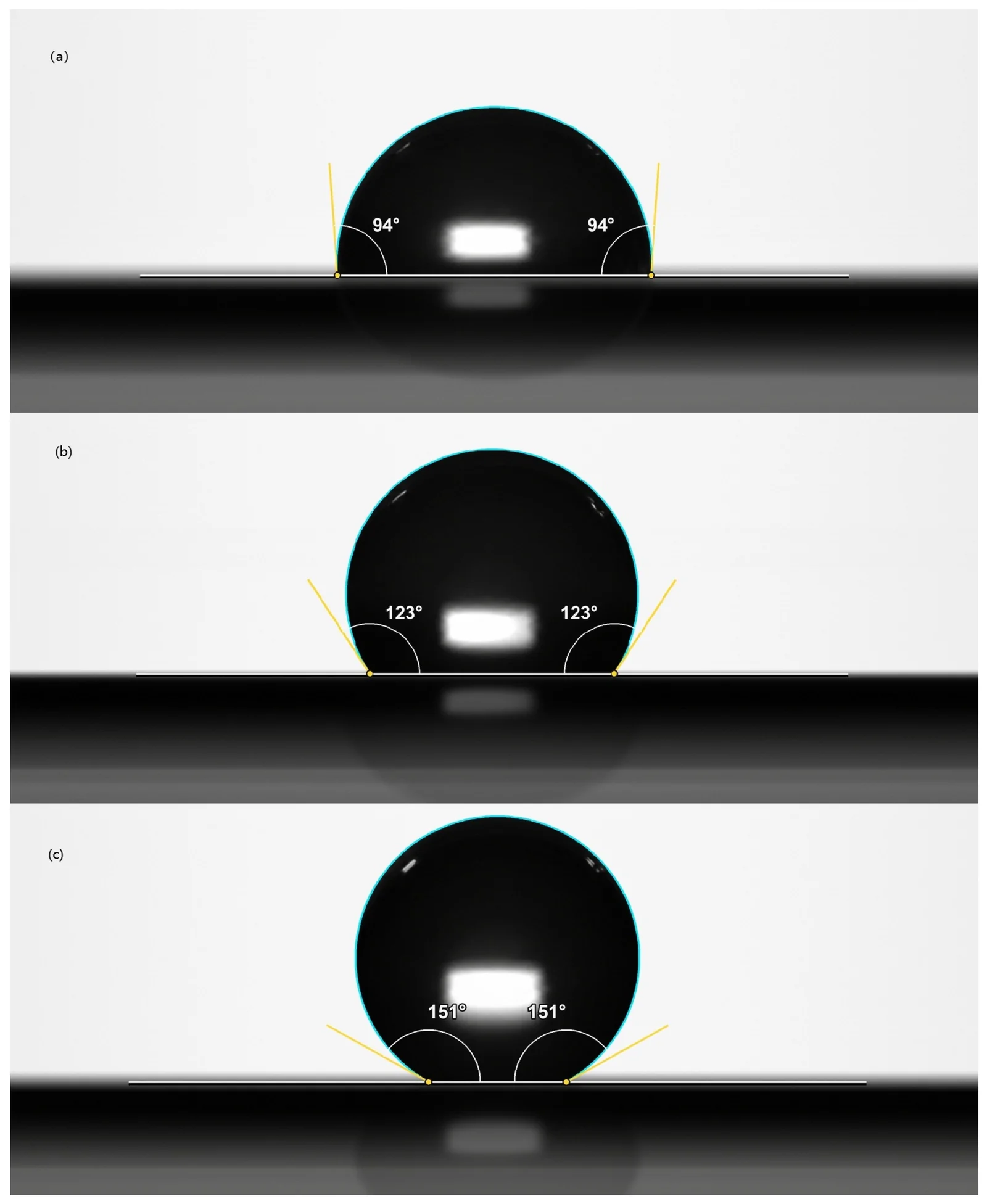
Representative sessile-drop profiles for MWCNT:FEP ratios: (**a**) 0:1, 94°; (**b**) 1:20, 123°; and (**c**) 1:10, 151°.

Surface-wetting measurements used 2 µL water droplets in the central DEG region, where the impact and maximum wetting occurred. The single archived apparent static contact angles were 94°, 123°, and 151° for 0:1, 1:20, and 1:10 MWCNT:FEP, respectively. Figure 5 retains the instrument baseline, both tangent constructions, and the left/right values. One retained sliding-angle measurement for 1:10 gave 6° at the same droplet volume; 38 µL was too large for this measurement. Peripheral measurements were not used as substitutes for central-region repeats. The values are descriptive and were not analysed inferentially; future work should repeat central measurements on independently prepared specimens.

Electrical output was evaluated under the matched droplet and circuit conditions described below. One device was used for each surface, and 100 peak-detection events were recorded per surface. The retained-water photograph was perspective-corrected using the visible corners of the 40 mm × 40 mm active surface. Visible liquid footprints were segmented manually, while the foreground object and lower edge were excluded. The central estimate was 26% of the unobscured surface, equivalent to approximately 420 mm^2^ if the same coverage is assumed over the full active area.

The same-process, FEP-only control substantially improved the comparison by holding spraying, primer, and thermal treatment constant. However, increasing MWCNT loading can change electrical conductivity and effective capacitance at the same time as wettability, so the voltage trend cannot be attributed to contact angle alone.

For a two-component Cassie–Baxter estimate, the apparent contact angle *θ*_CB_, reference FEP angle *θ*_ref_, and nominal projected solid fraction *f*_s_ are related by Equation (1) [25,26,27,28]. The complementary nominal air fraction is *f*_g_ = 1 − *f*_s_. The estimate assumes a composite interface and is used only as an interpretive descriptor; it is not a direct microscopic-area measurement.

(1) cos *θ*_CB_ = *f*_s_(cos *θ*_ref_ + 1) − 1

### 2.4. Droplet Generation and Environmental Conditions

UK mains tap-water droplets with a volume of 38 ± 1 µL were delivered by a peristaltic pump at 4 droplets s^−1^ (0.25 s between droplets). The needle-to-impact distance was approximately 100 mm. Droplets impacted the centre of the DEG so that the expanding liquid contacted the copper-wire electrode at maximum spreading. Ambient temperature was maintained at 22 ± 2 °C and relative humidity at 50%. The device was inclined at 30° relative to the horizontal. The same batch of tap water was used throughout. The droplet volume, release geometry, impact position, inclination, and interval were held constant across the compared conditions.

The nominal pre-impact Weber number was 82 and was calculated from the total incident droplet velocity using the conventional inertia-to-capillarity definition [31,32] given in Equation (2). The droplet volume, pump setting, release geometry, and needle-to-impact position were held constant across all wetting configurations; accordingly, the same nominal pre-impact Weber number was assigned to all configurations.

(2) We = *ρv*^2^*D*/*γ*

In Equation (2), *ρ* is the liquid density, *v* is the total pre-impact velocity, *D* is the droplet diameter, and *γ* is the liquid surface tension. The incident velocity was obtained from droplet displacement between consecutive frames recorded at 1000 frames s^−1^ before contact. The impact condition is, therefore, defined by the camera-derived incident velocity and Weber number, rather than by a free-fall estimate from the approximate release distance, because the droplet had an initial release velocity. Wetting can change spreading, contact-line motion, and residence after impact, but those post-contact dynamics do not alter the stated pre-impact Weber number definition and were not used to recalculate the We.

In all partial-immersion experiments, the droplet impact zone remained above the reservoir water line and did not contact the reservoir directly.

### 2.5. Wetting-State Protocols

Four conditions were evaluated in the wetting-position experiments. The blank control was tested without deliberate pre-wetting. For bottom wetting, the FEP film and ITO-coated glass electrode were brought together while fully submerged so that a continuous liquid film formed across their interface, as shown schematically in Figure 1b. The assembled pair was then removed from the bath; visible free droplets on exposed outer surfaces and edges were removed, while the unmetered interfacial film retained by the assembled interface remained in place at the time of droplet impact. Its volume was not measured. For top wetting, the polymer was immersed in water for 10 s and tested approximately 1 min after removal.

For the wetting-and-drying condition, the previously wetted sample was placed under a hot-air gun set to 120 °C, with the nozzle positioned 15 cm from the FEP, for 2 min. The endpoint was the absence of visually apparent surface liquid. The value of 120 °C is the hot-air-gun setting, not a measured FEP surface temperature. The sample was allowed to cool for approximately 10 min until it had returned to room temperature and was then mounted in the fixture; mounting required approximately 1 min.

### 2.6. Verification of Drying State

Drying was verified by the absence of visually apparent liquid and a gravimetric check after cooling to room temperature. The device assembly was weighed before wetting and after drying using a balance with a readability of 0.1 mg; the displayed post-drying mass returned to its pre-wetting value. This indicates that no retained mass was detectable at the balance resolution but cannot exclude sub-resolution moisture. The FEP surface temperature and post-drying contact angle, sliding angle, morphology, and surface chemistry were not measured. Accordingly, ‘wetted-and-dried’ denotes the state produced by the fixed 120 °C-setpoint/2 min protocol, and the associated voltage result is described as electrical recovery under that protocol rather than proof of unchanged surface properties or general restoration of function.

### 2.7. Partial-Immersion Protocol

A separate experiment evaluated immersion extents of 0%, 10%, 30%, and 50%, defined by the submerged length of the 40 mm DEG, which are shown in Table 1 along with all the other groups that will be tested. The corresponding water levels were measured and marked on the device before testing. The droplet-impact region remained completely above the reservoir water line while the submerged length was varied. The top-wetted condition is a different wetting configuration and was not treated as equivalent to 100% immersion.

### 2.8. Electrical Measurements

Voltage was acquired with an RS PRO RSDS1204CFL digital oscilloscope using a 10× voltage probe connected directly across an external 1.0 MΩ resistor (±1%). The oscilloscope input remained in its high-impedance mode throughout. No direct instantaneous-current instrument was used. For each device in the replicated wetting and immersion series, 100 events were stored at 0.01 s intervals in the peak-detection mode, with one droplet delivered every 0.25 s. Representative transient traces were sampled at 40 µs.

### 2.9. Power, Energy, and Charge Calculations

Following the established resistive-load treatment of DEG voltage waveforms [1,8,14], the current, instantaneous power, dissipated energy, and transported charge in the 1.0 MΩ external-resistor branch were calculated from Equations (3)–(6), respectively.

(3) *I*_R_(*t*) = *V*_R_(*t*)/*R*_L_

(4) *P*_R_(*t*) = [*V*_R_(*t*)]^2^/*R*_L_

(5) *E*_R,drop_ = ∫ *P*_R_(*t*) d*t* = (1/*R*_L_) ∫ [*V*_R_(*t*)]^2^ d*t*

(6) *Q*_R,drop_ = ∫ *I*_R_(*t*) d*t* = (1/*R*_L_) ∫ *V*_R_(*t*) d*t*

Equations (3)–(6) describe only the external 1.0 MΩ resistor branch and do not require the DEG capacitances to be approximated as short circuits. Capacitive impedance is frequency-dependent, and the voltage probe and oscilloscope input add parallel resistance and capacitance. The external-load current is obtained directly from Equation (3), whereas the total source current also contains the probe–oscilloscope branch current and cannot be recovered without its frequency-dependent impedance. Although the probe and oscilloscope settings were held constant, wetting-dependent changes in the DEG source impedance or effective capacitance could change voltage division and current partition between the two branches. The derived resistor-branch quantities, therefore, characterise the measured loaded response and are not invariant-load estimates of intrinsic source power across wetting states.

### 2.10. Statistical Analysis

The wetting and immersion families were analysed separately against their respective controls. Each condition comprised four separately assembled devices and 100 ordered droplet events per device. The device was the experimental unit: each device contributed the average of its 100 droplet cases, while droplets were treated as nested technical repeats. The group values are reported as the average of four device averages ± the between-device sample SD, with two-sided 95% *t* confidence intervals (df = 3). Because between-device variances could differ, Welch’s one-way ANOVA was used as the primary omnibus test. Targeted two-sided Welch comparisons evaluated bottom wetted, top wetted, and wetted-and-dried against the blank control, top wetted against bottom wetted, and each immersion level against the not-immersed control. The *p*-values were adjusted within each family using Holm’s method; the accompanying difference intervals are unadjusted 95% Welch intervals. The significance threshold was *α* = 0.05. The averages for the first and second halves of each 100-case sequence (50 cases per half) were compared descriptively to assess within-run ordering. Before analysis, all 3200 event files were checked for completeness, duplicate numbering, metadata consistency, time-axis continuity, and agreement between extracted peaks and oscilloscope labels; all passed. The earlier apparatus series was compared descriptively and not pooled, and the coating series remained a one-device-per-formulation analysis.

## 3. Results

### 3.1. Effect of Wetting Location and Drying

As shown in Table 2, across four devices per condition, the blank-control average was 166.55 ± 1.28 V (95% confidence interval, 164.51–168.59 V). The corresponding averages were 166.05 ± 2.24 V for the bottom-wetting (162.49–169.61 V), 72.93 ± 1.72 V for the top-wetting (70.19–75.66 V), and 167.18 ± 1.05 V for the wetted-and-dried state (165.50–168.85 V). Welch’s ANOVA showed a condition effect, *F*(3, 6.47) = 2748.33, *p* = 2.03 × 10^−10^.

Relative to the blank control, bottom wetting changed the average by −0.50 V or −0.30% (95% difference interval, −3.86 to 2.86 V; Holm-adjusted *p* = 0.960), so no appreciable suppression was detected at the present precision. Top wetting reduced the average by 93.63 V or 56.21% (−96.30 to −90.95 V; adjusted *p* = 2.62 × 10^−9^). The wetted-and-dried average was 0.625 V or 0.38% above the blank (−1.42 to 2.67 V; adjusted *p* = 0.960), indicating electrical recovery under the stated protocol. The displayed post-drying mass also returned to its pre-wetting value at 0.1 mg readability, although formal electrical equivalence and complete dehydration below that resolution were not established. Top wetting was also 93.13 V below bottom wetting (−96.64 to −89.61 V; adjusted *p* = 7.21 × 10^−9^).

The group-averaged values for the first and second halves of each 100-case sequence (50 cases per half) were 166.25 versus 166.85 V for the blank control (+0.36%), 165.77 versus 166.33 V for bottom wetting (+0.34%), 72.66 versus 73.19 V for top wetting (+0.73%), and 167.22 versus 167.14 V for the wetted-and-dried condition (−0.05%). The small changes lacked a common direction and were minor relative to the top-wetting contrast.

**Figure 2:**
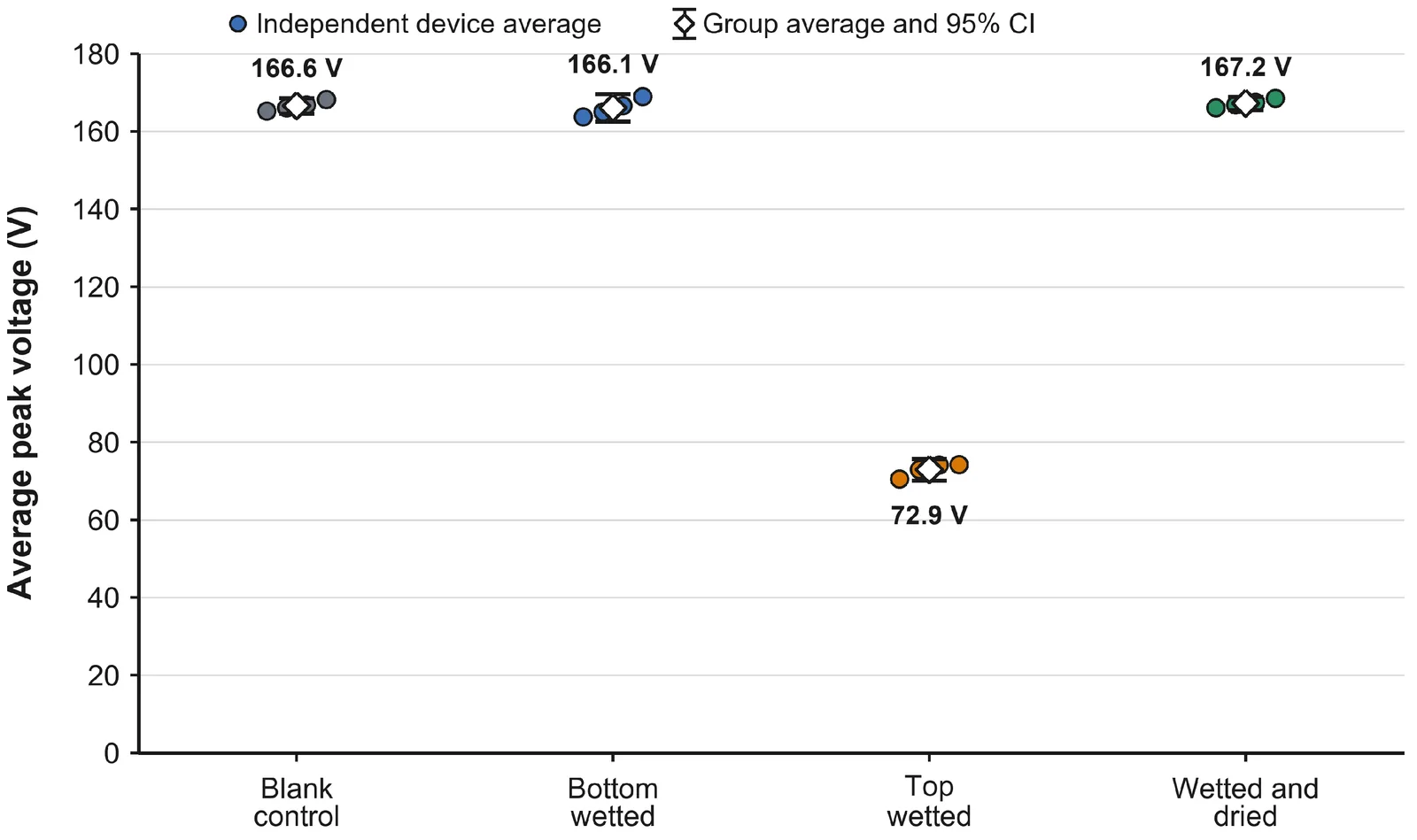
The device-level average peak voltage under the four wetting conditions. The circles are the device averages; the diamonds and bars are group averages and 95% confidence intervals (*n* = 4; 100 events/device).

Figure 2 displays all four device averages for each condition together with the group average and the between-device 95% confidence interval. The inferential sample size is four devices per condition, not the 400 nested droplet events.

### 3.2. Effect of Partial Immersion

As shown in Table 3, across four devices per condition, the average peak voltage decreased from 150.85 ± 5.85 V at 0% immersion (95% confidence interval, 141.54–160.16 V) to 121.73 ± 4.56 V at 10% (114.47–128.98 V), 98.68 ± 2.96 V at 30% (93.96–103.39 V), and 78.85 ± 1.98 V at 50% (75.69–82.01 V). Relative to the not-immersed control, the reductions were 19.31%, 34.59%, and 47.73%, respectively.

Welch’s ANOVA showed an immersion-condition effect, *F*(3, 6.23) = 210.95, *p* = 1.20 × 10^−6^. The differences from the not-immersed average were −29.13 V at 10% (95% difference interval, −38.33 to −19.92 V; Holm-adjusted *p* = 2.99 × 10^−4^), −52.18 V at 30% (−60.93 to −43.42 V; adjusted *p* = 1.17 × 10^−4^), and −72.00 V at 50% (−80.87 to −63.13 V; adjusted *p* = 1.17 × 10^−4^). These comparisons establish differences from the control; adjacent immersion levels were not tested inferentially.

The group-averaged values for the first and second halves of each 100-case sequence (50 cases per half) were 150.64 versus 151.06 V at 0% immersion (+0.28%), 121.79 versus 121.66 V at 10% (−0.11%), 98.94 versus 98.41 V at 30% (−0.54%), and 78.95 versus 78.76 V at 50% (−0.24%). No common directional ordering effect was evident.

### 3.3. Measured Immersion–Response Relationship

Across the measured 0–50% submerged-length range, the group average decreased monotonically, with a changing slope between the four measured conditions. The first 10% of the submerged length reduced the average by 19.31%, whereas increasing the submergence from 30% to 50% produced a further 13.14 percentage-point loss relative to the 0% average. These observations and the control comparisons do not define an asymptote, demonstrate a mathematical nonlinear law, establish differences between every adjacent level, or predict the response beyond 50% immersion.

**Figure 3:**
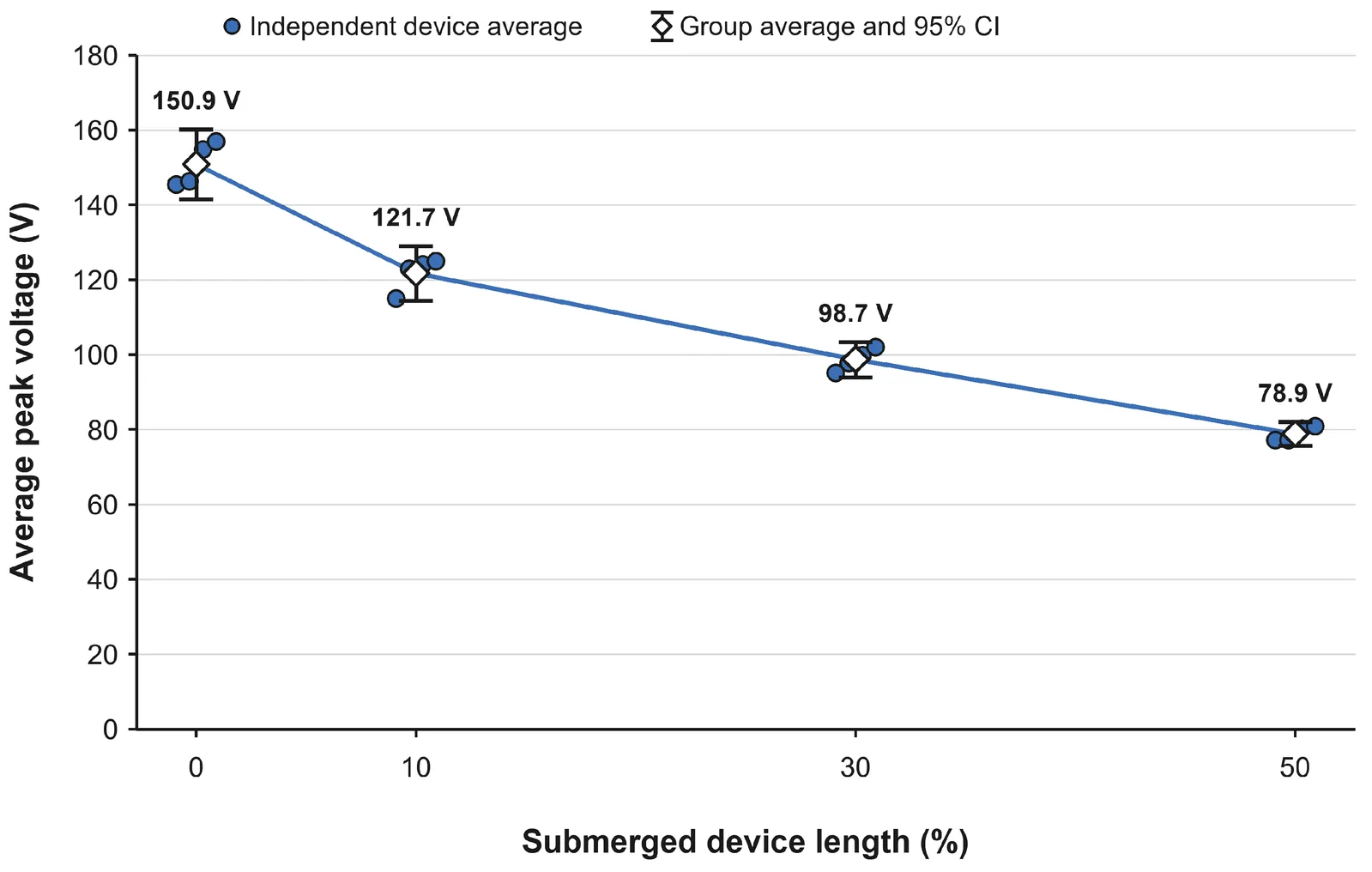
Device-level average peak voltage versus submerged device length. Circles are device averages; diamonds and bars are group averages and 95% confidence intervals (*n* = 4; 100 events/device).

In Figure 3, the circles represent the four device averages at each measured immersion extent, the diamonds and error bars show the group average and between-device 95% confidence interval, and the connecting line is a visual guide rather than a fitted model. The impact region remained above the reservoir water line throughout.

As a descriptive cross-apparatus check, the common-condition averages in the earlier repeat series differed from the present series by +0.35%, −1.49%, and −0.03% for the bottom-wetted, top-wetted, and wetted-and-dried states, respectively, and by 0.00%, +0.27%, +0.66%, and +1.45% across the 0%, 10%, 30%, and 50% immersion states. The two series were not pooled because they were acquired as separate experimental batches.

### 3.4. Representative Transient Response

Five recovered high-resolution voltage pulses had measured peaks of 74–92 V with matching 10× physical-probe and channel factors. Their full widths at half maximum were 0.16–0.20 ms, and the voltage fell from the peak to 10% within 0.24–0.28 ms. These differences are comparable to the 40 µs sampling interval, supporting a cautious statement that the main voltage-pulse duration was similar across these representative events despite amplitude variation.

An earlier raster current figure reports a calculated peak of approximately 213 µA, but it cannot be traced to the same raw voltage event and is inconsistent with Equation (3) for the documented 1.0 MΩ external load. It was, therefore, excluded from the quantitative power calculation. For the five recovered voltage pulses, the derived peak external-resistor current was 74–92 µA.

**Figure 4:**
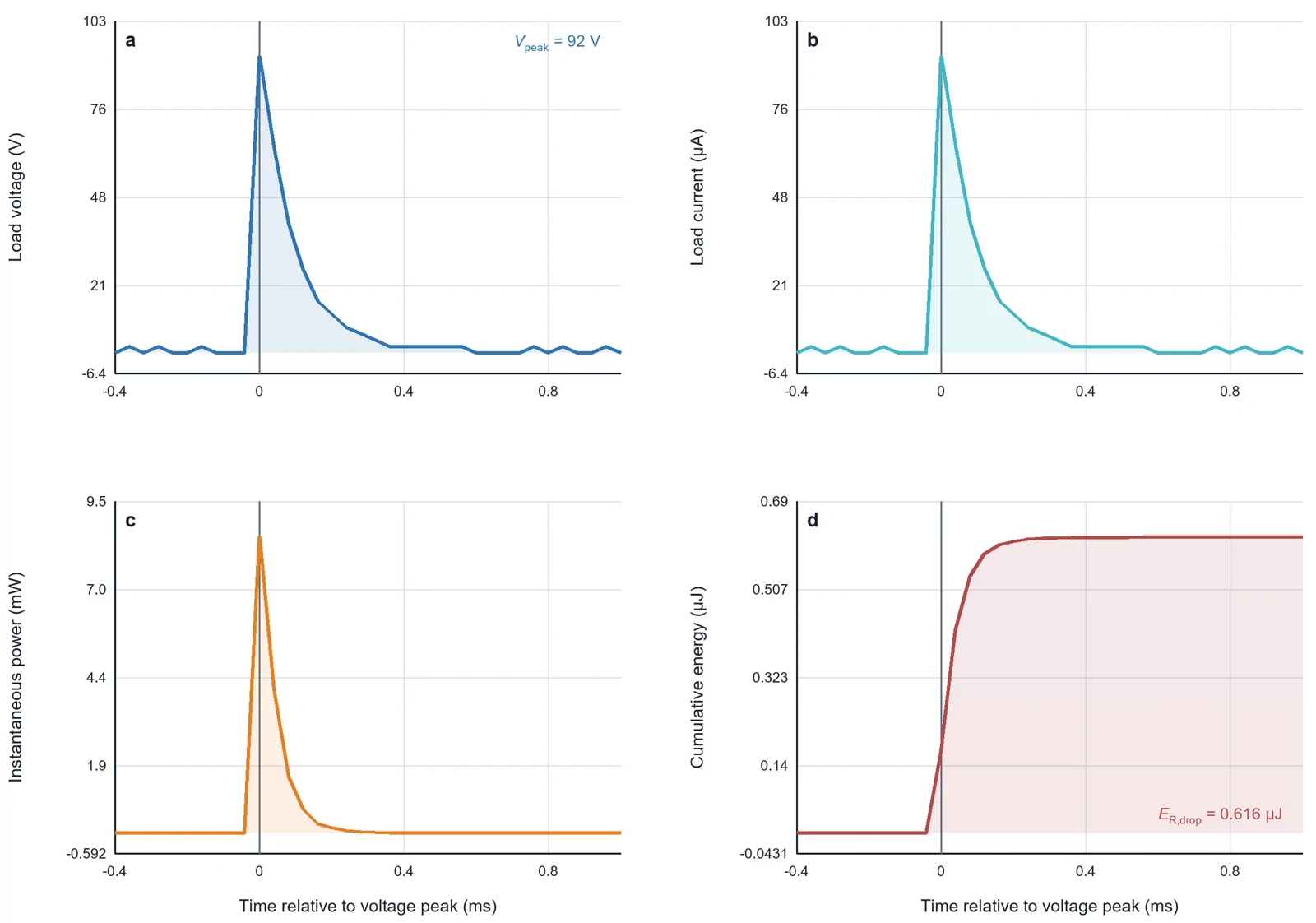
Representative external 1.0 MΩ resistor-branch transient: (**a**) voltage; (**b**) current; (**c**) power; and (**d**) cumulative energy.

The representative event plotted in Figure 4 reached 92 V, 92 µA, 8.464 mW, and 0.616 µJ in the external-resistor branch over the stated integration window; the parallel probe–oscilloscope branch is excluded.

### 3.5. Power and Energy Output

For the external 1.0 MΩ resistor branch only, using the voltage measured directly across that resistor (±1%) with matching physical-probe and channel factors of 10×, five high-resolution pulses gave derived peak branch currents of 74–92 µA (average 82.4 µA), peak branch powers of 5.476–8.464 mW (average 6.837 mW), and resistor-dissipated energies of 0.460–0.616 µJ per pulse (average 0.513 µJ) over a common −0.4 to +1.0 ms window relative to the voltage peak. These values characterise representative loaded pulses and are not used for cross-wetting comparisons of intrinsic generated power. The 10× probe–oscilloscope input forms an additional parallel branch; if wetting changes the DEG impedance, the current division and loading effect can also change. Resolving that dependence requires impedance or capacitance measurements under each hydration state.

### 3.6. Preliminary Low-Adhesion Target-Surface Comparison

Within the process-matched, spray-coated series, the MWCNT:FEP powder ratios of 0:1, 1:20, and 1:10 had representative apparent static contact angles of 94°, 123°, and 151°, respectively, as shown in Figure 5. The 1:10 formulation also had a measured sliding angle of 6° with a 2 µL droplet, meeting the stated low-adhesion target under that measurement condition. For the single device used for each formulation, the retained aggregate average peak voltages were 95.6, 90.8, and 83.5 V; these correspond descriptively to 95.0% and 87.3% of the FEP-only average for the 1:20 and 1:10 devices. Event-level uncertainty cannot be reconstructed from the retained aggregate source for this auxiliary series.

**Figure 6:**
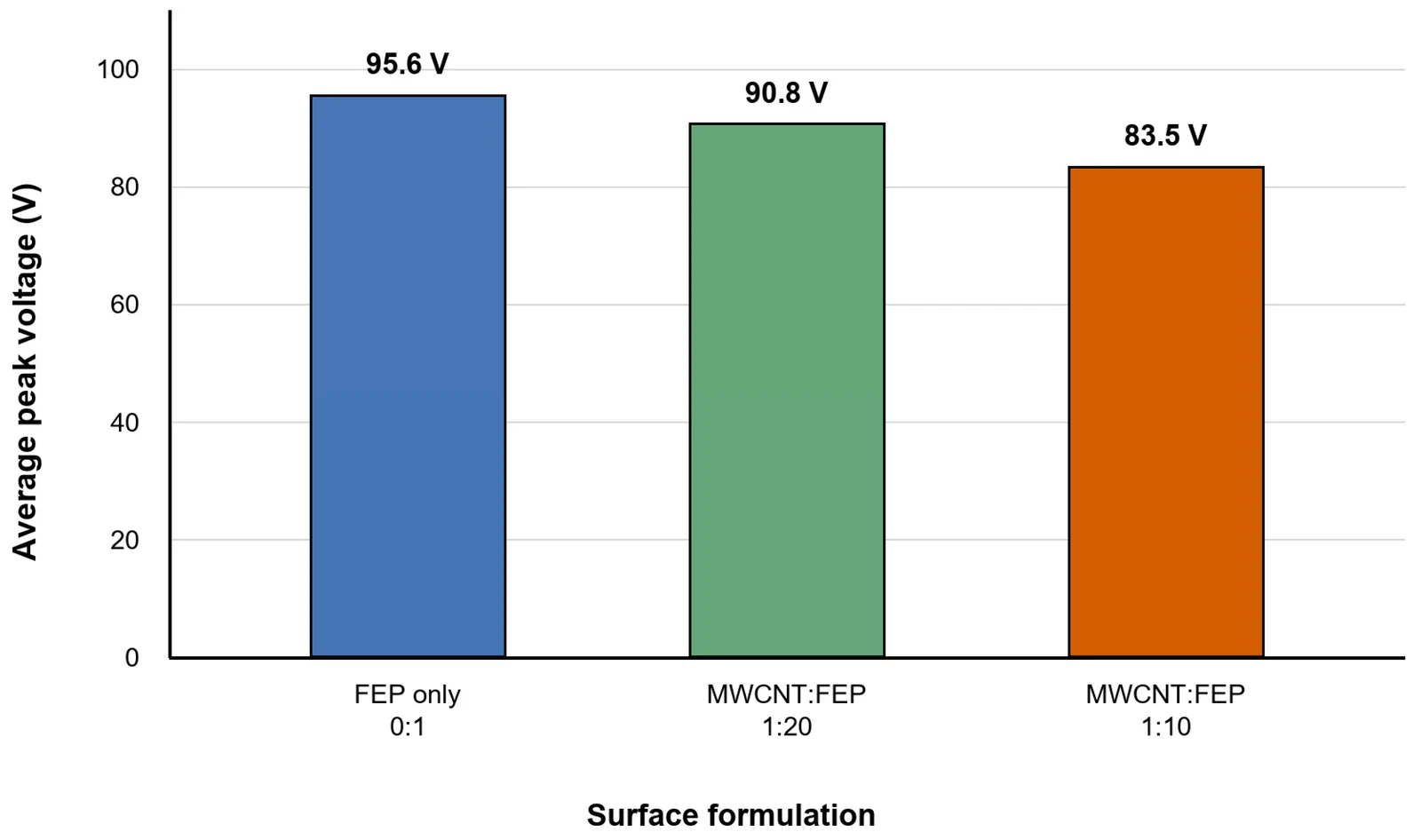
Average peak voltage for process-matched MWCNT:FEP coating formulations (100 droplet events; one device per formulation).

Using the process-matched 94° FEP-only apparent angle as *θ*_ref_ and the 151° apparent angle as *θ*_CB_ in Equation (1) gives a nominal solid fraction *f*_s_ of 0.135 and air fraction *f*_g_ of 0.865. This 86.5% nominal air fraction is an approximate Cassie–Baxter interpretation, not a direct measurement of microscopic contact area, because *θ*_ref_ is itself an apparent angle and dynamic droplet impact may partially penetrate or compress the texture [25,26,27,28]. Despite the large nominal reduction in projected solid–liquid contact, the average peak voltage decreased by only 12.7%, from 95.6 V for the process-matched FEP-only coating to 83.5 V for the 1:10 coating. The electrical response is, therefore, not proportional to the nominal Cassie solid fraction. Possible contributions include dynamic wetting of the texture, trapped-air compression, changes in interfacial charge density or effective capacitance, and MWCNT conductive pathways. These effects cannot be separated by the present data. Direct dynamic contact-area imaging, impedance measurements, and load-matched time-domain tests are required; Figure 6 reports peak voltage rather than maximum extractable power.

## 4. Discussion

### 4.1. Wetting Location Determines Response Magnitude

Across four devices per condition, wetting location produced a sharply asymmetric response. Bottom wetting changed the average by only −0.30% relative to the blank control, with a difference interval spanning zero and an adjusted *p*-value of 0.960. In contrast, top wetting reduced the average by 56.21% (adjusted *p* = 2.62 × 10^−9^). Top water directly changes the pre-impact condition at the active droplet–FEP interface, so effective contact geometry, local charge transfer, ionic screening or leakage, surface-potential distribution, and relaxation may all differ from the blank state [9,10,11,12,13,15,16,17].

The top-wetted average was also 93.13 V below the bottom-wetted average (adjusted *p* = 7.21 × 10^−9^), identifying the exposed surface as the location of greatest observed sensitivity under these conditions. Bottom-interface water was separated from the incoming droplet by 0.20 mm FEP; any effect through buried capacitance, field distribution, or leakage was, therefore, absent, too small, or too variable to resolve in the present geometry. This bounds rather than eliminates buried-interface mechanisms. Surface-potential, impedance, capacitance, synchronised charge, and impact-geometry measurements are still required to distinguish competing explanations.

### 4.2. Recovery After Drying

The wetted-and-dried group average was 100.38% of the blank-control average. The estimated difference was +0.625 V (95% interval, −1.42 to 2.67 V; adjusted *p* = 0.960). This near-agreement indicates electrical-output recovery under the specified 120 °C-setpoint/2 min protocol, but a non-significant difference is not a formal equivalence result. The displayed post-drying mass returned to its pre-wetting value at 0.1 mg readability, supporting the absence of gravimetrically detectable retained mass. This check cannot exclude moisture below the balance resolution or establish an unchanged FEP surface because post-drying contact angle, sliding angle, morphology, and surface chemistry were not measured.

### 4.3. Immersion Extent and Measured Changing-Slope Decline

The replicated immersion response in Figure 3 shows that water outside the immediate impact region can influence measured peak voltage: the averages were 19.31%, 34.59%, and 47.73% below the not-immersed control at 10%, 30%, and 50% submerged length, respectively, and each control contrast remained statistically supported after Holm adjustment. Because the reservoir never contacted the impact zone, the decline cannot be attributed to direct replacement of the top droplet–FEP interface. A physically consistent interpretation is that the conductive reservoir changes water-to-ground capacitance, electric-field distribution, and parasitic leakage in the coupled device–measurement system [3,6,8,9,14]. The changing slope also shows that submerged length is not a simple proxy for active contact area or an isolated circuit element. The connecting line guides the eye only; adjacent-level differences and responses beyond 50% were not tested.

### 4.4. Mechanistic Hierarchy and Evidential Boundary

Supported by the present data: Across four devices per condition, top-surface wetting produced a 56.21% reduction in loaded peak voltage, whereas no appreciable bottom-wetting suppression was detected at the present precision. The average voltage decreased monotonically across the measured immersion levels, and the wetted-and-dried average returned to within 0.38% of the blank-control average under the stated protocol. The small and inconsistently directed first-/second-half changes do not explain these condition contrasts. These observations show that wetting changes the coupled interfacial and electrical boundary, but they do not identify which microscopic or circuit parameter changed.

A testable working hierarchy is, therefore, proposed. Top-surface water is hypothesised to dominate because it directly changes the pre-impact state of the active droplet–FEP interface and can alter effective contact, screening, leakage, and charge relaxation. Bottom-interface water remains separated from the droplet by 0.20 mm FEP; its lack of a detectable response in this experiment suggests that buried capacitance, field redistribution, or leakage effects were comparatively weak under the tested geometry, not that they are impossible. Remote reservoir water may modify a much larger electrostatic and water-to-ground boundary, consistent with the replicated immersion trend. This hierarchy is a falsifiable interpretation, not identification of a unique mechanism; impedance spectroscopy between impacts, hydration-controlled capacitance, time-resolved surface-potential measurement, and synchronised impact imaging would discriminate among alternatives.

Not resolved by the present data: The measurements do not isolate the EDL formation, EDL area, charge density, effective contact area, ionic screening, leakage, charge-relaxation time, hydration-dependent dielectric properties, or impact/contact-line geometry. Electron transfer, ion adsorption, EDL formation, interfacial relaxation, electrostatic induction, and circuit loading may coexist at liquid–polymer interfaces [10,11,12,13,15,16,17]. Consequently, EDL formation is not presented as the sole or proven mechanism, and electrical recovery under the drying protocol is not treated as proof of complete moisture removal or unchanged surface chemistry.

### 4.5. Biomimetic Design Target and Evidential Boundary

The measured 94° contact angle shows why static hydrophobicity alone is an incomplete design criterion: a surface can be nominally hydrophobic while still allowing retention, pinning, or a persistent film. The lotus leaf motivates a low-adhesion target characterised by a small sliding angle and rapid departure at a modest tilt [19,20,21,22,23,24,25,26,27,28]. The sprayed 1:10 MWCNT:FEP sample met this limited functional target through a representative 151° apparent static contact angle and a 6° sliding angle for a 2 µL droplet, while its descriptive average peak voltage was 87.3% of the process-matched FEP-only value. Without morphology and quantitative multi-drop residue measurements, this is not evidence of lotus-like hierarchy, self-cleaning, or residue-free shedding.

Because the three formulations used the same spraying, primer, and thermal history, the increasing contact angle can be associated with MWCNT loading more confidently than in an unmatched coating comparison. However, MWCNT loading also changes conductive pathways and potentially the effective capacitance; the decline from 95.6 to 83.5 V, therefore, cannot be assigned to wettability alone.

### 4.6. Practical Significance and Environmental Operating Envelope

The practical role of this work is to identify when a laboratory-optimised DEG may lose output because its assumed dry initial state is not maintained. Relevant applications include rain-energy harvesters, self-powered rain or wetness sensors, splash-exposed monitoring nodes, and partially inundated infrastructure [1,2,3,4,5,6,7,8,9,18,19,20,21]. At 38 µL and four droplets per second, the localised delivery rate was 0.547 L h^−1^. If this volume were hypothetically distributed uniformly over the 40 mm × 40 mm active area, it would correspond to 342 mm h^−1^; however, the experiment used a localised droplet train and was not a calibrated rainfall simulation. No application prototype or field rainfall threshold is claimed.

Wetting effects are expected to become important when the 0.25 s inter-drop interval is shorter than drainage and evaporation times, under sustained intense rain or splashing, at high humidity that slows drying, at orientations that reduce the gravitational component driving droplet departure, or when poor sealing permits bottom-interface wetting or partial immersion [18,19,20,21,25,26,27,28]. The present tests establish the effect only at 22 ± 2 °C, 50% relative humidity, and a 30° device inclination. The 1:10 coating’s 6° sliding angle was measured with a 2 µL droplet and indicates a water-shedding margin relative to the 30° inclination, but it does not by itself predict performance for 38 µL impacts or natural rainfall. Rain intensity, humidity, and orientation should, therefore, be mapped independently in future field-oriented validation.

### 4.7. Limitations

- Device replication and inference: Each main wetting and immersion condition was evaluated using four separately assembled devices, and device averages were used as the experimental units. This provides basic between-device replication, but *n* = 4 remains modest; confidence intervals are consequently broadest where between-device variance is larger, and claims are bounded to the tested apparatus, material specification, fabrication protocol, and environmental conditions.
- Nested event sampling: The 100 droplets per device characterise within-device repeatability and ordering but do not constitute 400 independent specimens per condition.
- **Acquisition:** Peak-detection data support peak-voltage comparisons but do not retain full energy waveforms for every droplet.
- Power measurement: All derived current, power, charge, and energy values refer only to the external 1.0 MΩ resistor branch. The parallel probe–oscilloscope branch is excluded, and wetting-dependent source-impedance changes may alter current division; no intrinsic cross-wetting source-power comparison is claimed.
- Surface characterisation: Contact angles and the 1:10 coating’s 6° sliding angle (2 µL) are single descriptive central-region measurements; independently prepared specimens were not tested. Peripheral positions were not substituted because the functional impact/wetting region is central. Sliding angles for the other formulations, morphology, quantitative residue data, and event-level coating-series intervals are unavailable. Drying verification was limited to the return of the displayed post-drying mass to its pre-wetting value at 0.1 mg readability; post-drying contact/sliding angles, surface chemistry, and morphology were not measured.
- **Immersion range:** The available series ends at 50% submerged length. It supports a monotonic decline over the measured range but does not define the response at full immersion or establish an asymptotic plateau.
- Biomimetic claim: The 1:10 MWCNT:FEP surface is presented only as a lotus-inspired low-adhesion target based on its apparent static contact angle and 2 µL sliding angle. Lotus-like hierarchical morphology, self-cleaning, and residue-free shedding were not verified, and independently replicated residue reduction remains to be demonstrated.

## 5. Conclusions

Across four separately assembled devices per condition, the response to pre-existing water depended strongly on location and extent. Top wetting reduced the average peak voltage by 56.21% relative to the blank control, whereas bottom wetting changed it by −0.30% and no appreciable suppression was detected at the present precision. Increasing the submerged length to 10%, 30%, and 50% reduced the average by 19.31%, 34.59%, and 47.73%, respectively, over the measured range.

Following the stated drying protocol, the wetted-and-dried group average was within 0.38% of the blank-control average, and the displayed post-drying mass returned to its pre-wetting value at 0.1 mg readability. Together, these findings support electrical-output recovery with no gravimetrically detectable retained mass under that protocol, but do not establish formal equivalence, complete dehydration below the balance resolution, unchanged surface properties, or general reversibility. The replicated top-versus-bottom contrast supports a location-dependent working hierarchy, but the effective contact area, EDL properties, ionic screening, leakage, charge relaxation, dielectric hydration, and impact geometry remain unisolated. Four devices per condition provide basic between-device replication, while the modest sample size and single apparatus/material protocol continue to limit broad generalisation.

In the process-matched coating series, the 1:10 MWCNT:FEP formulation reached a representative apparent static contact angle of 151° and a 6° sliding angle for a 2 µL droplet, while its descriptive average peak voltage was 87.3% of the FEP-only average. This supports compatibility with a preliminary lotus-inspired low-adhesion target, but it does not demonstrate lotus-like hierarchy or residue-free shedding. MWCNT electrical effects, quantitative multi-drop residue reduction, surface morphology, event-level uncertainty for the retained aggregate series, and between-device repeatability of the coating comparison remain unresolved.

## Figures and Tables

**Table 1:** The experimental conditions used in the wetting-state study.

Condition	Interfacial State	Treatment	Role	Devices; Events/Device
Blank control	No intentional pre-wetting	Untreated device	Reference	4; 100
Bottom wetted	Liquid beneath polymer	Liquid layer at polymer–bottom-electrode interface	Buried-interface test	4; 100
Top wetted	Pre-wetted active surface	FEP soaked for 10 s, then removed	Surface-wetting test	4; 100
Wetted-and-dried	Previously wetted; dried	Heat gun at 120 °C for 2 min	Recovery test	4; 100
Partial immersion	0%, 10%, 30%, 50% submerged length	Marked water levels; impact region remained dry	Extent-response test	4 per level; 100

**Table 2:** A device-level peak-voltage summary for the wetting conditions.

Condition	*n*	Events/Device	Average ± SD (V)	95% CI (V)	Change vs. Blank (%)	Adjusted *p*
Blank control	4	100	166.55 ± 1.28	164.51–168.59	0.00	—
Bottom wetted	4	100	166.05 ± 2.24	162.49–169.61	−0.30	0.960
Top wetted	4	100	72.93 ± 1.72	70.19–75.66	−56.21	2.62 × 10^−9^
Wetted-and-dried	4	100	167.18 ± 1.05	165.50–168.85	+0.38	0.960

**Table 3:** A device-level peak-voltage summary for the immersion conditions.

Submerged Length (%)	*n*	Events/Device	Average ± SD (V)	95% CI (V)	Reduction (%)	Adjusted *p*
0	4	100	150.85 ± 5.85	141.54–160.16	0.00	—
10	4	100	121.73 ± 4.56	114.47–128.98	19.31	2.99 × 10^−4^
30	4	100	98.68 ± 2.96	93.96–103.39	34.59	1.17 × 10^−4^
50	4	100	78.85 ± 1.98	75.69–82.01	47.73	1.17 × 10^−4^

**Figure A1:**
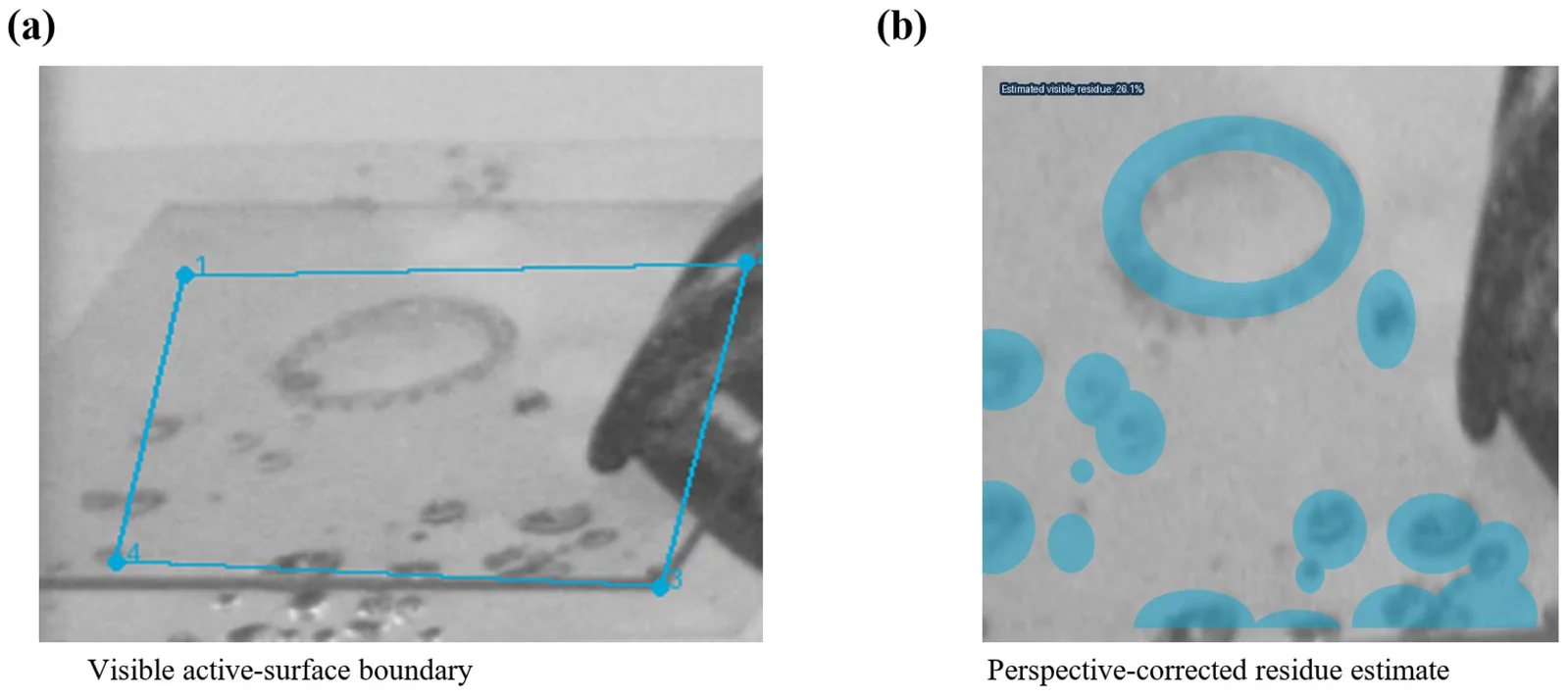
Preliminary retained-water image analysis: (**a**) the original oblique photograph with the visible active-surface boundary; and (**b**) the perspective-corrected segmentation used to estimate the visible water-covered area.

## Data Availability

Data will be made available on request.

## Nomenclature

The following mathematical symbols are used in this manuscript:

*α*_s_	Sliding angle (°)
*γ*	Liquid surface tension (N m^−1^)
*η_V_*	Retained average-voltage ratio (%)
*θ*_s_	Static water contact angle (°)
*ρ*	Liquid density (kg m^−3^)
*A*_active_	Electroactive surface area (mm^2^)
*A*_res_	Area covered by retained water (mm^2^)
CV	Coefficient of variation (%)
*D*	Droplet diameter (m)
*E*_R, drop_	Energy dissipated in the external load per droplet (J)
*f*_res_	Retained-water area fraction (%)
*I*_R_(*t*)	Instantaneous current in the external load (A)
*P*_R_(*t*)	Instantaneous power dissipated in the external load (W)
*Q*_R, drop_	Charge transported through the external load per droplet (C)
*R*_L_	External load resistance (Ω)
*s_V_*	Sample standard deviation of peak voltage (V)
*t*	Time (s)
*v*	Droplet impact velocity (m s^−1^)
$V_{peak}^{-}$	Average peak voltage (V)
*V*_R_(*t*)	Voltage measured across the external load (V)
We	Weber number (dimensionless)
*f*_g_	Nominal Cassie air fraction (dimensionless)
*f*_s_	Nominal Cassie projected solid fraction (dimensionless)
*θ*_CB_	Apparent contact angle of the composite Cassie–Baxter surface (°)
*θ*_ref_	Reference apparent contact angle of the process-matched FEP surface (°)

## Abbreviations

The following abbreviations are used in this manuscript:

CV	Coefficient of variation
DEG	Droplet-based electricity generator
EDL	Electric double layer
FEP	Fluorinated ethylene propylene
FWHM	Full width at half maximum
ITO	Indium tin oxide
MWCNT	Multi-walled carbon nanotube
SD	Standard deviation

## Appendix A. Preliminary Retained-Water Image Analysis

The available retained-water photograph was analysed to provide a transparent preliminary estimate of surface coverage. The visible corners of the 40 mm × 40 mm active surface were used for perspective correction. Water footprints were then segmented manually, while the foreground object and the partly obscured lower edge were excluded.

The retained-water fraction was defined for this image analysis using Equation (A1):

(A1) *f*_res_ = (*A*_res_/*A*_active_) × 100%

where *A*_res_ is the segmented retained-water area, and *A*_active_ is the 40 mm × 40 mm electroactive area (1600 mm^2^). Equation (A1) is an operational area metric defined for the present image analysis rather than a constitutive physical model.

The central estimate was 26% of the unobscured active surface. A defensible range of 20–30% is reported. If the visible fraction is extrapolated over the complete 1600 mm^2^ active area, the central estimate is approximately 420 mm^2^ and the reporting range is approximately 320–480 mm^2^. Because the experimental record does not identify the photographed surface formulation or the number of applied droplets, this estimate is presented only as an illustrative image-analysis result.
